# The Interpersonal-Psychological Theory of Suicide to Explain Suicidal Risk in Eating Disorders: A Mini-Review

**DOI:** 10.3389/fpsyt.2021.690903

**Published:** 2021-06-17

**Authors:** Patrizia Zeppegno, Raffaella Calati, Fabio Madeddu, Carla Gramaglia

**Affiliations:** ^1^Department of Translational Medicine, Institute of Psychiatry, Università del Piemonte Orientale, Novara, Italy; ^2^S.C. Psichiatria, Azienda Ospedaliero Universitaria Maggiore della Carità, Novara, Italy; ^3^Department of Psychology, University of Milan-Bicocca, Milan, Italy; ^4^Department of Adult Psychiatry, Nîmes University Hospital, Nîmes, France

**Keywords:** suicide, eating disorders, thwarted belongingness, perceived burdensomeness, acquired capability for suicide

## Abstract

Suicide is a major cause of death in Eating Disorders (EDs) and particularly in anorexia nervosa (AN). The aim of the present mini-review was to summarize the literature focusing on the interpersonal-psychological theory of suicide (IPTS) by Thomas E. Joiner, as applied to explain suicidal risk in EDs. PubMed database was used to search articles focused on IPTS in EDs; 10 studies were eventually included. The majority of the included studies reported data from the same sample, even though the hypotheses and analyses for each study were unique. The investigated suicidal outcomes were suicidal ideation (SI) (40%), non-suicidal self-injury (10%), suicide attempt (40%) and suicide (10%). In ED patients Perceived Burdensomeness (PB) may play an important role, especially regarding SI risk. ED patients may feel like a burden to their close ones, and actually some of the ED symptoms may be an expression of anger and hate against the self. Overall, currently available research has supported some IPTS derived predictions (i.e., ED symptoms may increase PB and thereby SI), but not others (i.e., the elevated suicide rate in AN may be due to higher acquired capability for suicide). Further research on IPTS tenets as well as on other theoretical perspectives and constructs (e.g., interoceptive awareness), hopefully with a longitudinal design and adequate follow-up duration, might allow a more thorough understanding of the complex topic of suicidal behavior in ED patients.

## Introduction

Every year 800,000 people die by suicide worldwide (1). Even though the phenomenon may be underestimated, suicide has been suggested to be a major cause of death in Eating Disorders (EDs) (2), and it is likely the first or second cause of death in patients with anorexia nervosa (AN) (2–5). Recently, also suicide attempts (SA) were found to be a major issue in EDs, especially in binge-purging subtypes, i.e., in bulimia nervosa (BN) (21%) and binge-purging AN (AN-bp) (25.6%) compared to restrictive AN (AN-r) (9–10%) (6).

Some shortcomings of the existing literature about the topic should be underscored (4, 5): the majority of the available research is cross-sectional or retrospective, which leaves the timing of the mortality risk unclear; virtually all research has been conducted using the Diagnostic and Statistical Manual of Mental Disorders-IV (DSM-IV) definitions, hence the impact of changes to ED diagnoses in DSM-5 on prevalence rates of suicidal behavior has still to be better understood. Last, the high rate of comorbid psychopathology and of diagnostic crossover in EDs may also have affected the reported relationships between EDs and suicidal behavior.

Despite these shortcomings, a higher frequency of suicide is usually found in AN (4), while a higher one of SA is found in BN (6). One hypothesis to explain this discrepancy may be based on the fact that, compared to BN, AN patients may be more compromised from a medical standpoint (3), hence it is possible that, in their case, a SA eventually leads to suicide, while it would not in “healthier” BN patients, notwithstanding the underlying intention to die. In any case, it is likely that the meaning of suicidal behavior is different in AN and BN. Indeed, it is more likely that for AN patients the desired outcome of a SA is death, as they usually show higher intent and lethality, similar to suicidal individuals. On the other hand, for BN patients SA may represent an expression of multi-impulsivity (7) or an attempt to achieve affect regulation. From this standpoint, the focus of a suicide-prevention approach should be on meaning in life for AN, and rather on affect regulation skills and impulsivity for BN (3, 4).

Some at-risk features for SA and suicide have been identified in ED patients, such as purging type, chronicity of disease, low Body Mass Index (BMI) for AN, comorbidity with major depression, obsessive symptoms, drug abuse (2–5). The role of major depression has been supported quite consistently across studies (2–6, 8), as the one of comorbid alcohol/drug abuse (2–6, 8, 9) and binge/purging subtype (2, 3, 6, 8). Affective problems and/or dysregulation (4–6, 8–10) and impulsivity (2, 6, 8–10) may be relevant, as well. Other factors include comorbid anxiety, comorbid cluster B personality disorders, obsessive traits, need for control, perfectionism, self-criticizing cognitive style, poor self-esteem, interoceptive deficits, trauma-related issues (2–6, 8–10).

Briefly, although it is acknowledged that EDs are associated with suicidal ideation (SI), SA, and suicide death, little is known about the dynamic interplay between these conditions. In other words, it is possible that EDs either directly or indirectly contribute to suicidality, as well as the reverse. It is also possible that EDs and suicidality share common biological and psychological dysfunctions that eventually lead a given individual to be more likely to experience both (5). Furthermore, a clear approach to suicidality in EDs through the lens of a specific theory of suicide is still lacking, even though suggestions have been proposed about the relationship between ED symptoms, death and self-inflicted death. The self-destructiveness and the constant attacks against the body which are implicit and typical in ED behaviors have to do with death, either with a drive toward it or with an all-powerful denial of it, in the struggle to exist within the narrowest parameter (11). Bruch underscored that as AN patients feel guilty for surrendering to the gross and vulgar demands of the body, they may choose and want to live as the self, but to die as the body (12). It has been argued that AN patients are not attracted by death, but rather they are seeking control over their life in the struggle to gain a sense of identity. Anyway, since they fail in really achieving such control, the ED symptoms represent a latent suicidal act as the result of feeling depressed, while maintaining an illusion of “false” control (12–17).

The Interpersonal-Psychological Theory of Suicide (IPTS), introduced by Thomas E. Joiner in 2005 (18), is aimed at explaining the differences in individual suicidal behaviors. The three constructs underlying the IPTS, which interact with each other, are the following (19): Thwarted Belongingness (TB) and Perceived Burdensomeness (PB) would predict SI, while the Acquired Capability (AC) for suicide would be linked to suicidal behavior. For a lethal SA, according to this theory, all three domains should be present; the fact that they are generally co-occurring only in a subgroup of individuals is the reason why the lifetime suicide rate is lower than that of ideation, which is present in 15% of the population (20, 21).

The TB construct describes a sort of “barrier” preventing some individuals to feel satisfied with their relationships, for the absence of support networks or because they do not feel a real connection with others, despite having frequent contacts. Two specific variables are present in TB: loneliness (e.g., to feel disconnected from others), and the absence of reciprocal care (e.g., neither to support nor to receive support from others). The PB construct describes a feeling of being so incompetent and unable to offer a meaningful contribution to the relationship and that one's existence represents a burden to anyone, to the point that her/his death has more value for others than her/his life. Two variables have been described also for PB: liability (e.g., the feeling that one's own death is worth more than the life to others) and self-hate (e.g., hate against the self). The AC construct is linked to the fact that some individuals, through a history of repeated painful experiences, are able to get used to the fear and pain involved in self-harm, becoming more fearless (if the fear actually diminishes), more courageous (if the fear persists but is tolerable) or both (18). AC includes two variables as well: fearlessness about death (FAD) and elevated physical pain tolerance.

TB and PB are assessed with the Interpersonal Needs Questionnaire (INQ) (22) while AC is assessed with the Acquired Capability for Suicide Scale (ACSS) (23).

The aim of the present mini-review was to summarize the literature findings where the IPTS was tested to explain suicidal risk in any ED.

## Methods

### Search Strategy

A literature search was performed to identify studies focusing on the IPTS in EDs. PubMed database was used to search articles using the following search terms: [(Joiner) OR (interpersonal theory of suicide) OR (thwarted belongingness) OR (perceived burdensomeness) OR (acquired capability) OR (capability for suicide) OR (fearlessness about death) AND (eating disorders)].

**Figure 1:**
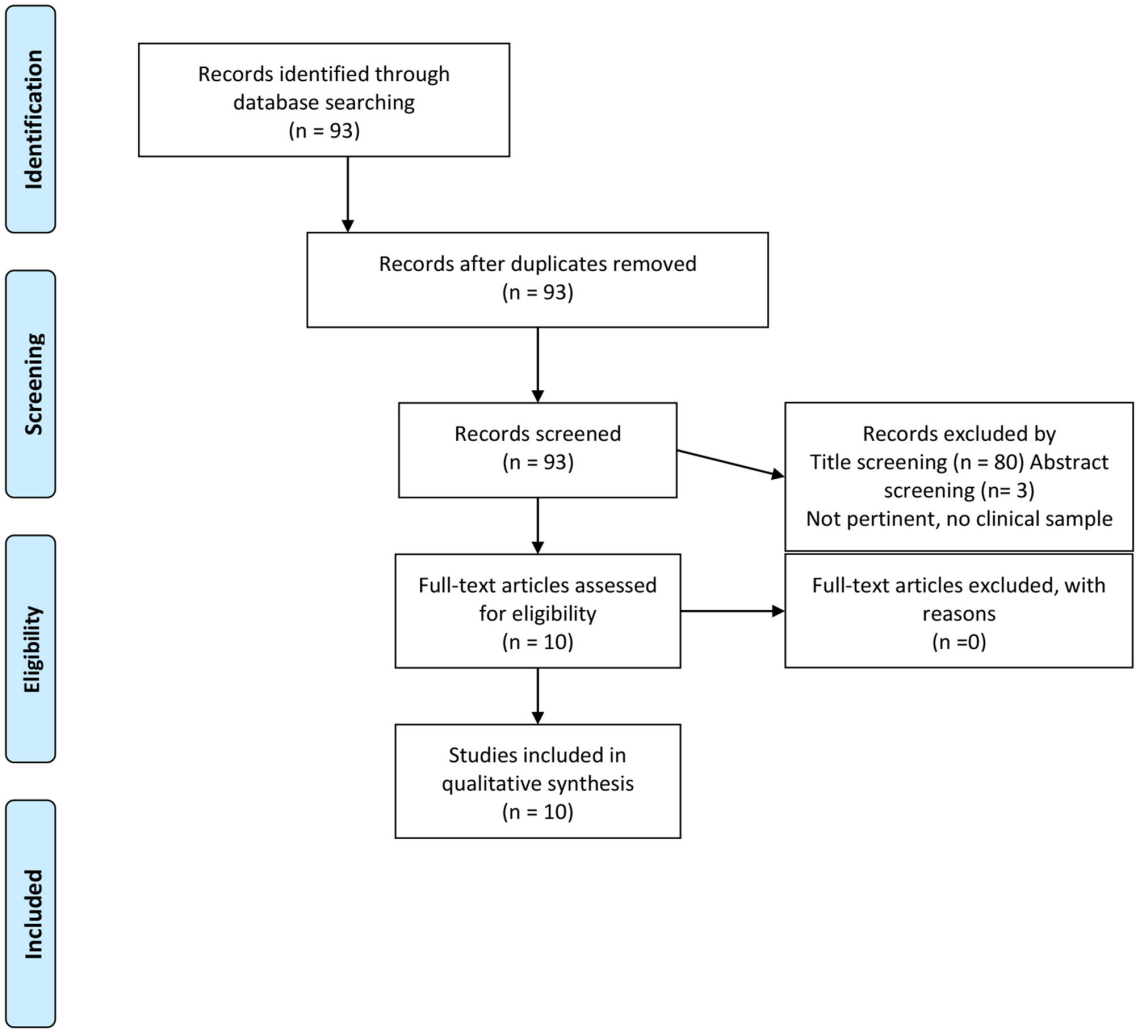
PRISMA 2009 flow diagram.

Following the Preferred Reporting Items for Systematic Reviews and Meta-Analyses (PRISMA) (24) flowchart, studies selection was made on February 28th 2021, screening titles first, then abstracts and eventually the full texts of the articles. Two independent reviewers (CG and RC) assessed the articles identified by the search string; a third reviewer (PZ) resolved any discrepancy that emerged between the reviewers. See Figure 1 for details.

Studies were included if (1) they examined any type of ED; (2) they focused on IPTS; (3) they focused on any form of suicide-related outcome: SI, non-suicidal self-injury (NSSI), SA, and suicide; (4) they were written in English.

Studies were excluded if: participants were not ED patients.

The reference lists of the identified studies and reviews were checked as well for further relevant articles.

The following data were extracted and tabulated: first author name and year of publication, country, study design, main aim, suicidal outcomes, sample features (such as gender, age, BMI, diagnosis), methods (scales), and main findings.

Study quality was assessed, as appropriate, with the Strengthening the Reporting of Observational Studies in Epidemiology (STROBE) (25) and with the Quality Assessment Tool for Case Series Studies (26).

## Results

Figure 1 shows the study selection procedure: titles, abstracts and full texts were excluded in case they were not pertinent to the review topic or did not assess a clinical sample. Table 1 includes the data extracted from the 10 studies we eventually included in the mini-review (27–33).

**Table 1:** Main features of the studies included in the mini-review (listed in alphabetical order).

**Study**	**Country**	**Design**	**Aim**	**Suicidal outcome**	**Sample features**	**Methods**	**Main findings**
Bodell et al. (29)	USA	Naturalistic longitudinal study	To examine between- and within-person associations between burdensomeness, belongingness and SI	SI	97 females (*N* = 78 residential treatment; *N* = 17 partial hospitalization) DSM-5 diagnoses: *N* = 33 AN *N* = 27 BN *N* = 29 OSFED *N* = 1 BED *N* = 7 USFED Mean age 26.7 ± 7.6	Baseline assessment: EDE-Q; BDI-II Weekly assessment across 12 weeks of treatment: DSI-SS; INQ	Patients with higher levels of perceived burdensomeness reported higher mean symptoms of SI. Neither between- nor within-person effects of belongingness were associated with SI. Levels of burdensomeness, but not thwarted belongingness, significantly predicted SI at the subsequent week. SI itself predicted burdensomeness.
Dodd et al. (31)	USA	Cross-sectional	To assess the association between interoceptive deficits, NSSI and SA. To investigate the role of ACS facets (FAD and pain tolerance) as links in the association between NSSI and SA, and between interoceptive deficits and SA	NSSI, SA	*N* = 96 ED patients DSM-5 diagnoses: *N* = 34 AN *N* = 27 BN *N* = 35 OSFED Mean age 26.8 ± 7.9 *N* = 70 previous NSSI *N* = 26 at least one lifetime SA	EDI-3 Interoceptive Deficits subscale FASM ACSS FAD subscale Subjective pain tolerance (one Likert-type item)	Significant association between interoceptive deficits and NSSI; between interoceptive deficits and SA; between interoceptive deficits and FAD. Significant association between NSSI and both FAD and pain tolerance. Significant association between previous SA and pain tolerance, but not FAD. Indirect relation between interoceptive deficits and SA; largely mediated by NSSI, FAD and pain tolerance. Limited support for IPTS-derived hypotheses.
Forrest et al. (33)	USA	Cross-sectional	To determine whether current and lifetime ED symptoms were positively related to SI through thwarted belongingness and perceived burdensomeness in ED patients	SI	*N* = 100 ED patients from residential (*N* = 80) or partial hospitalization ED treatment DSM-5 diagnoses: *N* = 34 AN *N* = 27 BN *N* = 30 OSFED *N* = 1 BED *N* = 8 USFED Mean age 26.92 ± 7.86	EDE-Q EDI-3 Body Dissatisfaction Scale EPPES BDI-II DSI-SS INQ	First model (current symptoms): current body dissatisfaction and fasting were related (indirectly) to increased SI through higher burdensomeness (controlling for depression). Second model (lifetime symptoms): lifetime fasting was related (indirectly) to increased SI through higher burdensomeness (controlling for depression). Current and lifetime ED symptoms, as body dissatisfaction and fasting, may increase burdensomeness. Body dissatisfaction and fasting were positively related to thwarted belongingness, which anyway was neither a significant positive predictor of SI nor a robust mediator between ED symptoms and SI.
Holm-Denoma et al. (27)	USA and Germany	Case reports (9 cases)	To investigate reasons for the occurrence of death by suicide in AN, in the light of Joiner's theory of suicide	Death by suicide	Sample 1: *N* = 4 cases of death by suicide in AN patients from the USA; mean age: 24.8 Sample 2: *N* = 5 cases of death by suicide in AN patients from Germany	Examination of 9 case reports of patients died by suicide in a sample of patients followed for AN. Sample 1: SCID SADS ED-LIFE Sample 2: SIAB Focus on likelihood of methods to result in death and likelihood of being rescued.	Explanation of high rates of suicide in AN: use of highly lethal methods (8 of the 9 cases) in the context of low rescue potential (7 of the 9 cases). Use of highly lethal methods is in line with Joiner's theory of SB, especially fearlessness about death. Convergent support for Joiner's hypothesis about the link between AN and a relatively high rate of suicide.
Pisetsky et al. (36)	US	Cross-sectional	To test the Interpersonal Theory of Suicide (IPTS) in ED	SI, SA	*N* = 114 ED AN 8.8% BN 21.1% BED 23.7% ED NOS 46.5% 93.9% female Age: 33.7 ± 12.11 88.6% in outpatient treatment, 11.4% in day treatment or residential	INQ PPES ACSS-FAD EDE-Q	65 participants (57.0%) had lifetime SI. 24 (21.1%) had lifetime SA. Thwarted belongingness and perceived burdensomeness were associated with lifetime SI. Painful and provocative events were associated with lifetime SA.
Selby et al. (28)	Multi-site study across North America and Europe	Cross-sectional	To explore whether repetitive exposure to painful and destructive behaviors such as vomiting, laxative use, and NSSI was a mechanism that linked AN-binge-purging (ANBP) subtype, as opposed to AN-restricting subtype (ANR), to extreme suicidal behavior	SA	Study 1: *N* = 787 AN Age: 29.7 ± 11.2 Study 2: *N* = 249 AN Age: 26.30 ± 8.50	EATATE SIAB DIGS	Study 1: Structural equation modeling results supported provocative behaviors as a mechanism linking ANBP to suicidal behavior. A second, unexpected mechanism emerged linking ANR to suicidal behavior via restricting. Study 2: Replicated findings of Study 1, including the second mechanism linking ANR to SA. Two potential routes to suicidal behavior in AN seem to have been identified: one route through repetitive experience with provocative behaviors for ANBP, and a second for exposure to pain through the starvation of restricting in ANR.
Smith et al. (32)	US	Cross-sectional, Case-control	To test the Interpersonal Theory of Suicide (IPTS) in ED	SI, SA	*N* = 100 ED *N* = 85 Psychiatric patients *N* = 93 College students	INQ ACSS-FAD DSI-SS	Within the ED sample, no interaction was found, but perceived burdensomeness was associated with SI, and perceived burdensomeness and fearlessness about death were associated with past SA. The ED and psychiatric patients had greater thwarted belongingness, perceived burdensomeness, and SI than college students.
Trujillo et al. (34)	US	Longitudinal, Cohort	To examine the bidirectional, longitudinal relationship between ED symptoms and thwarted belongingness and perceived burdensomeness	–	*N* = 92 ED treatment-seeking 95.6 female Age: 32.82 ± 11.99	EDDS-5 EDI INQ	T1 ED symptoms did not predict T2 TB or PB. T1 TB did not predict T2 ED symptoms. T1 PB did predict T2 ED symptoms. Among participants with AN/sub/AN, T1 TB and PB predicted T2 ED symptoms.
Velkoff and Smith (35)	USA	Longitudinal study (8 weeks)	To examine between-person variability inn within-person change in ACS in ED patients over the course of 8 weeks of treatment	ACS	*N* = 100 ED patients from residential facility DSM-5 diagnoses: *N* = 34 AN *N* = 27 BN *N* = 30 OSFED *N* = 1 BED *N* = 8 USFED Mean age 26.92 ± 7.86 *N* = 27 at least one previous SA *N* = 45 SI at baseline *N* = 77 previous NSSI	Weekly assessments with the ACSS FAD subscale and subjective pain tolerance (as assessed by one Likert-type item) (number of assessments = 8.17 ± 5.5)	Patients had midlevel ACS at baseline. Growth mixture modeling found no significant linear change in any of the two facets of ACS (FAD and pain tolerance) over the course of treatment. ACS may be more stable than originally theorized.
Witte et al. (30)	USA	Cross-sectional	To test the hypothesis that the extreme restrictive eating (characteristic of AN) facilitates acquiring the capability for suicide	SA	*N* = 100 ED female patients 26.92 ± 7.86 (range: 18–58) Primarily non-Hispanic (96%) and White (94%)	ACSS-FAD EDE-Q Physical pain tolerance	Findings did not support Joiner's hypothesis that restrictive eating is key in acquiring the capability for suicide.

*Diagnoses acronyms: AN, Anorexia Nervosa; AN-BP, Anorexia Nervosa binge/purging type; AN-R, Anorexia Nervosa restricting type; BED, Binge Eating Disorder; BN, Bulimia Nervosa; ED NOS, Eating Disorder Not Otherwise Specified; OSFED, other specified feeding or eating disorder; USFED, unspecified feeding or eating disorder. Suicidal behavior acronyms: ACS, Acquired capability for suicide; NSSI, Non-suicidal self-injury; SA, suicide attempt; SB, suicidal behavior; SI, suicidal ideation. Questionnaires acronyms: ACSS, Acquired Capability for Suicide Scale; ACSS-FAD, Acquired Capability for Suicide Scale – Fearlessness About Death; BDI, Beck Depression Inventory; DIGS, Diagnostic Interview for Genetics Studies; DSI-SS, Depressive Symptom Index–Suicidality Subscale; EATATE, Eatate-life Phenotype; EDDS-5, Eating Disorder Diagnostic Scale for DSM-5; EDE-Q, Eating Disorder Examination Questionnaire; EDI, Eating Disorder Inventory; ED-LIFE, Eating Disorders Longitudinal Interval Follow-up Evaluation; EPPES, Eating Behaviors Painful and Provocative Events Scale*.

Most of the included studies reported data from the same sample from a larger study, even though the hypotheses and analyses for each study were unique (29–33, 35).

Eight out of the 10 studies were performed exclusively in the US; 2 (20%) involved samples recruited both in the US and in another country [Germany, for (27); Europe for (28)].

Study design was cross-sectional in 6 (60%) out of 10 studies (28, 30–33, 36); it was longitudinal in 3 (30%) studies only (29, 34, 35). The remaining study was a case series (27).

All the studies included ED patients only (27–31, 33–36), except for the one by Smith et al. (32) which included a control group of psychiatric patients and a control group of college students.

Sample size ranged from a minimum of 9 patients in the case series about suicide death by Holm-Denoma et al. (27) to a maximum of 787 AN patients in the study by Selby et al. (28).

Regarding suicidal outcomes, the studies investigated the following: SI (40%) (29, 32, 33, 36); NSSI (10%) (31); SA (40%) (28, 31, 32, 36); death by suicide (10%) (27). In the remaining studies, the outcome was specifically related to IPTS dimensions, for instance the study by Trujillo et al. (34) focused on TB and PB and the ones by Velkoff and Smith and Witte et al. (30, 35) on AC.

Most studies included at least one measure for EDs, usually one of the Eating Disorders Inventory (EDI) versions or the Eating Disorders Examination Questionnaire (EDE-Q); this datum was not specified in some studies (32, 35). Regarding measures for the IPTS, the INQ was used in the following studies: (29, 33, 34) while the ACSS was used in these ones: (30, 31, 35). Some studies used both the INQ and the ACSS (32, 36). Holm-Denoma et al. (27) studied 9 cases of suicide in AN patients and Selby et al. (28) analyzed two samples of AN patients in the light of the IPTS, even though they used no specific measure.

The results from the studies involving the same sample were the following (29–33, 35): patients with higher levels of PB, but not TB, also reported more SI-related symptoms; furthermore, a bi-directional relation between SI and PB was found, as PB predicted SI at the subsequent week, while SI predicted PB (29); the IPTS hypothesis that restrictive eating might be key in AC was not supported (30); significant associations were found between pain tolerance and both NSSI and previous SA, and between FAD and NSSI, but not SA (31); PB was associated with SI, while both PB and FAD were associated with previous SA (32); both current (body dissatisfaction and fasting) and lifetime (fasting) ED symptoms were indirectly related to SI through higher PB, after controlling for depression (33); in an 8-week longitudinal study, no significant linear change in any of the two facets of AC (FAD and pain tolerance) was reported, leading the Authors to conclude that AC could be a more stable construct than originally supposed (35).

Holm-Denoma et al. with their case series including 9 deaths by suicide in AN patients supported Joiner's hypothesis about a link between AN and a relatively high suicide rate, as they found a use of highly lethal methods in the face of a low rescue potential, in line with the IPTS assumptions, especially those about FAD (27). Pisetsky et al. found an association of both TB and PB with lifetime SI, and a further association of painful and provocative events with lifetime SA (36). Selby et al. found two possible pathways to suicidal behavior, especially SA, in AN (28): one through repetitive experience with provocative behaviors (vomiting, laxative use, NSSI) in the binge/purging subtype of AN, and one through the painful experience of starvation in the restricting subtype. Trujillo et al. studied the associations between ED symptoms and TB and PB (34). ED symptoms at baseline did not predict either TB or PB at follow-up. Baseline TB did not predict ED symptoms at follow-up, while baseline PB did.

Tables 2, 3 describe the study quality assessment performed with the Strengthening the STROBE, except for the Holm and Denoma study (24) which was evaluated with the Quality Assessment Tool for Case Series Studies.

**Table 2:** Strengthening the Reporting of Observational Studies in Epidemiology (STROBE) scores of the included studies.

**N**	**Study**	**Title and abstract**		**Introduction**		**Methods**														**Results**											**Discussion**				**Other information**
		**1**		**2**	**3**	**4**	**5**	**6**		**7**	**8**	**9**	**10**	**11**	**12**					**13**			**14**			**15**	**16**			**17**	**18**	**19**	**20**	**21**	**22**
		**a**	**b**					**a**	**b**						**a**	**b**	**c**	**d**	**e**	**a**	**b**	**c**	**a**	**b**	**c**		**a**	**b**	**c**						
1	Bodell et al. (29)	1	1	1	1	1	1	1	NA	1	1	0	1	1	0	1	1	0	0	1	0	0	0	1	1	1	NA	NA	NA	1	1	1	1	1	1
2	Dodd et al. (34)	1	1	1	1	1	0	0	NA	1	1	1	1	1	1	0	1	NA	1	0	0	0	1	1	NA	1	0	NA	NA	1	1	1	1	1	0
3	Forrest et al. (33)	1	1	1	1	1	1	1	NA	1	1	0	1	1	1	1	1	NA	0	1	0	0	1	1	NA	1	NA	NA	NA	1	1	1	1	0	0
4	Pisetsky et al. (2016)	0	1	1	1	1	0	1	NA	1	1	0	0	1	1	1	0	NA	0	1	0	0	1	0	NA	1	NA	NA	NA	1	1	1	1	0	0
5	Selby et al. (28) Study 1	0	1	1	1	1	0	1	NA	1	1	1	0	1	1	1	1	NA	1	0	0	0	1	0	NA	0	NA	NA	NA	1	1	1	0	0	1
	Selby et al. (28) Study 2					0	0	1	NA	1	1	1	0	1	1	1	1	NA	1	0	0	0	0	0	NA	0	NA	NA	NA	1	1	1	1	1	
6	Smith et al. (32)	1	1	1	1	1	1	1	1	1	1	0	0	0	1	1	1	0	0	1	0	0	1	0	NA	1	NA	NA	NA	1	1	1	1	1	0
7	Trujillo et al. (34)	0	1	1	1	1	1	1	NA	1	1	0	0	1	1	1	1	0	0	1	0	0	1	0	1	1	NA	NA	NA	1	1	1	1	1	0
8	Velkoff and Smith (35)	1	1	1	1	1	0	0	NA	1	0	0	0	0	1	1	1	0	1	0	0	0	0	0	0	1	NA	NA	NA	1	1	1	1	1	1
9	Witte et al. (30)	0/NA	NA	1	1	1	0	0	NA	1	1	0	0	1	1	0	1	NA	1	1	0	0	1	0	NA	1	NA	NA	NA	1	1	1	1	1	0

*NA, not applicable*.

**Table 3:** Quality of reporting of the included case series study according to the Quality Assessment Tool for Case Series Studies.

**Study**	**1**	**2**	**3**	**4**	**5**	**6**	**7**	**8**	**9**	**Quality rating (Good, Fair, or Poor)**
	**Was the study question or objective clearly stated?**	**Was the study population clearly and fully described, including a case definition?**	**Were the cases consecutive?**	**Were the subjects comparable?**	**Was the intervention clearly described?**	**Were the outcome measures clearly defined, valid, reliable, and implemented consistently across all study participants?**	**Was the length of follow-up adequate?**	**Were the statistical methods well-described?**	**Were the results well-described?**	
Holm-Denoma et al. (27)	Yes	Yes	Yes	Yes	NA	Yes	Yes	NA	Yes	Good

*NA, not applicable*.

## Discussion

The aim of this mini-review was to summarize literature focusing on the IPTS by Thomas E. Joiner, to better understand the phenomenon of suicidal risk in EDs in the light of this theoretical model. From the perspective of the IPTS, it has been suggested that suicidal behaviors are frequent in EDs (in particular in AN), because ED behaviors, like dietary restriction, constitute painful and provocative experiences that could increase capability for suicide (4–6). In other words, EDs might indirectly increase risk for suicidal behavior in patients, via the AC for suicide.

The available evidence summarized in this mini-review failed to support a role for TB in the suicidal behavior of ED patients. Indeed, only the study by Pisetsky et al. (36) described an association between TB and SI. Thus, satisfaction with relationships does not seem to play a key role in suicidal behavior for ED patients. It is not clear whether this is due to the fact that patients are indeed satisfied with relationships, or to the fact that they do not consider relations a relevant issue. On the other hand, some evidence supported an association between PB and suicidal behaviors, either SI (29, 32, 33, 36) or SA (32). Therefore, in ED patients it seems that PB may play an important role, especially regarding SI risk. ED patients may feel incompetent and like a burden to their close ones, and actually some of the ED symptoms may be an expression of anger and hate directed against the self.

Symptoms like extreme fasting and starvation, vomiting and other purging behaviors, may be linked to self-hate and self-aggression and represent a sort of equivalent of self-injury; furthermore, they represent recurrent painful experiences, and according to the IPTS tenets they may eventually increase suicidal risk through AC for suicide (28). Indeed, even though elevated physical pain tolerance is consistent with the ED clinical picture (both in restricting and binge/purging ED subtypes), research findings are not consistent regarding elevated pain tolerance among those with AN and BN compared to those without EDs (28, 31). With more detail, FAD was associated with NSSI but not SA (31); nonetheless Holm-Denoma et al. considered their findings consistent with the IPTS assumptions about FAD, as the 9 suicide deaths they described in AN patients were characterized by the choice of highly lethal methods and by poor chances of rescue (27). This is in contrast with the “fragility hypothesis” according to which AN individuals would have a higher risk of suicide death because of their starvation-induced frailty (37). According to this theory, a non-lethal SA would become lethal for an AN subject. However, high lethal methods were found in AN as well (27).

Pain tolerance was associated with NSSI and previous SA (31) and, in line with this finding, painful provocative behaviors (such as purging ones and NSSI) and the painful experience of starvation were both considered possible pathways to suicidal behavior (28) and their association with lifetime SA was supported (36). On the contrary, Witte et al. did not support the role of the painful experience of restrictive eating in building AC (30).

Briefly, ED behaviors like vomiting, laxative use, and over-exercise may be associated with FAD (elements of AC for suicide) while other ED factors, like restriction and AN diagnosis, may not. Hence, study results are not conclusive regarding the construct of FAD, which does not seem higher than in psychiatric comparison groups (28).

Furthermore, regarding the AC dimension as composed by the two facets of FAD and pain tolerance, it was also suggested that it might be a much more stable construct than originally theorized, as no change was found over an 8-weeks period by Velkoff and Smith (35). Nonetheless, the dearth of longitudinal studies about this topic, and the brief period of observation of the available ones, do not allow to draw definitive conclusions.

To the best of our knowledge, this is the first mini-review focused on IPTS in EDs. Some limitations should be underscored, such as the limited number of included studies, the fact that many of them were performed in overlapping samples, which could represent a bias; the fact that all the available studies were performed in US, except for two which also involved samples from European Countries. Last, of course, considering the focus of this work, other theoretical approaches to suicidality in EDs have not been addressed.

Regarding studies' quality, the most critical issues emerging from the STROBE assessment were the following: the description of setting, location and relevant dates (item 5); the explanation of efforts to address possible sources of bias (item 9); details about how study size was arrived at, reason for non-participation at each study stage and number of participants with missing data (items 10, 13b, 13c, 14b). Last, most studies failed to acknowledge source of funding (item 22).

Summarizing, available research findings included in this mini-review only partially supported some of the IPTS tenets. Nonetheless, it has to be underscored that the IPTS was primarily developed to explain suicide deaths, which are not easy to address in scientific studies. Indeed, only the case series by Holm-Denoma et al. dealt with suicide deaths (27), while all the other studies included in this mini-review were about either SI or SA, or about specific IPTS constructs, which may represent a rather different situation. Furthermore, the available literature is mainly based on cross-sectional studies, which do not allow to understand the possible evolution of TB, PB and AC over time. Even though Velkoff and Smith found no change in AC (35), it might be argued that the assessment period could have been too short to highlight any change (just 8 weeks).

Further studies focusing on IPTS tenets as well as on other theoretical perspectives and constructs [e.g., interoceptive awareness, as in the Dodd et al. study (31)], hopefully addressing the critical issues emerged from the studies' quality assessment performed in the current mini-review, with a longitudinal design and adequate follow-up duration might offer a more thorough perspective on the complex topic of suicidal behavior in ED patients.

## Author Contributions

CG and PZ conceived the study. CG and RC performed the literature screening and review. Any discrepancy about reviewers was resolved with the consultation with PZ. The manuscript was drafted by CG and RC and revised for relevant intellectual content by PZ and FM. All the authors read and approved the final version of the manuscript.

## Conflict of Interest

The authors declare that the research was conducted in the absence of any commercial or financial relationships that could be construed as a potential conflict of interest. The handling editor declared past co-authorships with several of the authors, PZ and CG.
